# Thermal Control of Plasmonic Surface Lattice Resonances

**DOI:** 10.1021/acs.nanolett.1c04898

**Published:** 2022-05-04

**Authors:** Jussi Kelavuori, Viatcheslav Vanyukov, Timo Stolt, Petri Karvinen, Heikki Rekola, Tommi K. Hakala, Mikko J. Huttunen

**Affiliations:** † Photonics Laboratory, Physics Unit, Tampere University, FI-33014 Tampere, Finland; ‡ Faculty of Science and Forestry, Department of Physics and Mathematics, University of Eastern Finland, FI-80101 Joensuu, Finland

**Keywords:** metamaterials, plasmonics, surface lattice resonance

## Abstract

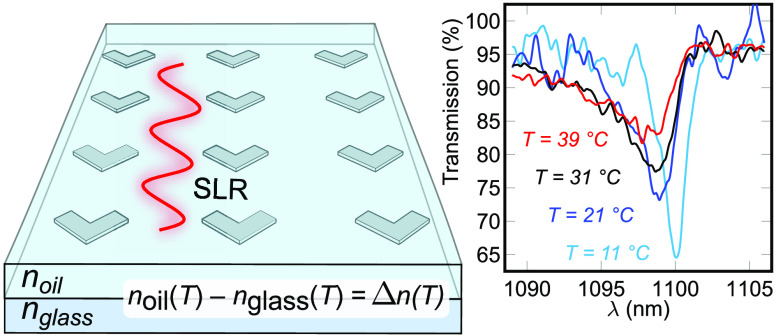

Plasmonic metasurfaces exhibiting collective responses known as surface lattice resonances (SLRs) show potential for realizing flat photonic components for wavelength-selective processes, including lasing and optical nonlinearities. However, postfabrication tuning of SLRs remains challenging, limiting the applicability of SLR-based components. Here, we demonstrate how the properties of high quality factor SLRs are easily modified by breaking the symmetry of the nanoparticle surroundings. We break the symmetry by changing the refractive index of the overlying immersion oil by controlling the ambient temperature of the device. We show that a modest temperature change of 10 °C can increase the quality factor of the SLR from 400 to 750. Our results demonstrate accurate and reversible modification of the properties of the investigated SLRs, paving the way toward tunable SLR-based photonic devices. More generally, we show how symmetry breaking of the environment can be utilized for efficient and potentially ultrafast modification of the SLR properties.

Optical metamaterials are artificial structures that allow control of light in ways not found in nature.^1^ Current research on optical metamaterials covers a wide spectrum of topics including saturable absorption,^2^ nanoscale phase engineering,^3^ epsilon-near-zero behavior,^4,5^ and supercontinuum generation.^6,7^ Furthermore, considerable interest has been focused on plasmonic metasurfaces consisting of metallic nanoparticles (NPs). Metallic NPs exhibit collective responses of conduction electrons known as localized surface plasmon resonances (LSPRs).^8^ On top of their high modifiability, the LSPRs increase the local near fields at the NP surface, enhancing the occurring light–matter interaction, making applications such as biosensing,^9^ lasing,^10^ and nonlinear optical processes^11^ possible. Unfortunately, metallic NPs suffer from high losses due to the ohmic nature of metals. The losses can be reduced by arranging NPs periodically and utilizing collective responses of periodic structures known as surface lattice resonances (SLRs).^12^ SLRs are diffractive–plasmonic hybrid resonances associated with high quality factors (*Q*-factors) that can reach values above 2000.^13^ Consequently, SLRs have already found several applications, including lasing^14^ and second-harmonic generation.^15,16^

Although SLR-supporting metasurfaces are easily designed and fabricated, their postfabrication control remains challenging. Earlier, control has been achieved, for example, by straining the elastic substrate of the metasurface,^17^ using refractory materials and raising the ambient temperature to very high values^18^ or by using optically^19^ or thermally^20^ responsive polymers. Furthermore, temperature-dependent optical responses of plasmonic waveguides^21^ and LSPRs^21−23^ have been already studied. However, no work has yet demonstrated an easy and universal approach to fine-tune the spectral positions of high-*Q* SLRs (*Q* ≥ 500). Such capabilities will nevertheless be needed in the future, when SLR-based devices will be utilized for applications where the wavelength of incident light cannot be changed, such as in modulation of wavelength-division multiplexed optical communication signals.

Here, we demonstrate dramatic control over the *Q*-factor and the extinction of a high-*Q* SLR by breaking the symmetry of the surroundings via temperature-dependent substrates. Unlike previous work, our devices operate close to room temperature, making our approach broadly applicable. We demonstrate how a decrease of 10 °C in the ambient temperature of the devices result in an increase of the *Q*-factor from 400 to 750, a 15% increase in the extinction, and a 1 nm shift in the center wavelength, λ_c_, of the SLR. Our results demonstrate accurate and reversible modification of the spectral properties of SLRs, paving the way toward tunable SLR-based photonic devices. More generally, our results demonstrate how highly efficient and potentially ultrafast tuning of SLR properties could be achieved by breaking the symmetry of the dielectric environment.

The properties of LSPRs are central in the field of plasmonics. The properties depend on the size, shape, environment, and the material of the NP.^8^ The LSPRs dictate the optical properties of plasmonic NPs, which can be understood by introducing the polarizability of a NP α and by connecting it to the induced dipole moment **p** and to the incoming field **E** via^8^
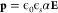
1where ε_0_ and ε_*s*_ are the vacuum and surrounding medium permittivities, respectively. The details of the calculation of the polarizability, α, are given in the Supporting Information.

LSPRs suffer from broad line widths and low *Q*-factors induced by the high ohmic losses typical for metals. The *Q*-factors of plasmonic metasurfaces have been successfully improved by arranging the NPs in periodic lattices and utilizing the emerging diffractive properties.^24^ In such structures, the diffraction modes can couple with the individual LSP modes, giving rise to collective responses known as SLRs.

The formation of SLRs can be calculated with a semianalytical method known as lattice-sum approach (LSA).^25^ In the LSA, the effects of scattered fields affecting the NPs are reduced to a single variable, called the lattice sum, *S*. In homogeneous environment, the lattice sum can be derived using the free-space dyadic Green’s tensor (see the Supporting Information for more information).

A NP near a planar interface between materials with different refractive indices show modified dipole radiation pattern with a large portion of the energy emitted into the optically denser material.^26^ This hinders the formation of the diffraction mode, and ultimately, the intensity and line width of the SLR.^27^ Under these circumstances, we can also expect the diffractive modes to form independently in each material, in which case the heterogeneous lattice sum can be estimated to be
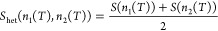
2where *S*(*n*) is the lattice sum in homogeneous environment, while *n*_1_ and *n*_2_ are the refractive indices of the substrate and superstrate, respectively. We note that *S*(*n*) associated with an optically denser environment would be expected to be slightly stronger than the term associated with a less dense medium. However, the weights for both environments were determined to be the same by comparing the shift in the peak location in our experiments to the LSA calculations. We suspect this discrepancy could be explained by the physical location of the NPs in the optically less dense medium (oil), which counteracts the effects of the modified dipole radiation pattern. The LSA uses *S* to modify α to include the effects of the scattered fields on a single NP. Once taken into account, the effective polarizability, α*, of a NP can be written as
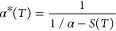
3Note that the inspection here is only valid for one polarization type. A tensorial approach is needed for more general results.^25^ Once α* has been solved, the transmission through the metasurface can be calculated using^28^
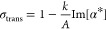
4where *A* = *p*_*x*_*p*_*y*_ is the area of the unit cell of the array, *p*_*x*_ and *p*_*y*_ are the periodicities of the NP array in respective dimensions, and *k* is the amplitude of the wave vector for light inside the superstrate (oil). We note that use of the unit cell area, *A*, should allow generalization of the treatment to particle arrays with non-orthogonal primitive lattice vectors.

When the diffractive modes form separately in the different materials, as estimated by eq 2, already small differences in the refractive indices of the materials can cause drastic changes in the *Q*-factors and extinction spectra of the measured devices. This occurs because the diffractive mode conditions shift to different wavelengths for the substrate and superstrate, consequently broadening the line width of the measured extinction peak.

The difference between the refractive indices of the substrate and superstrate is controlled in this work via temperature. In addition to affecting the environment, temperature has a small but undeniable effect also on the responses of individual NPs, which was investigated numerically in the Supporting Information. Although the small temperature changes used in this work were estimated to result in negligible changes in the optical responses of individual NPs, we note that it would be interesting to study whether cryogenic temperatures could facilitate realizations of ultrahigh-*Q* SLRs.

In this work, we investigated an array of V-shaped aluminum NPs with periodicities of 727 nm in the *y*-direction and 400 nm in the *x*-direction (see Figure 1). The NPs were 30 nm thick with 140 nm long and 70 nm wide arms. The NPs were fabricated using electron-beam lithography on a 1 mm thick D263T glass substrate from Schott. Olympus type-F immersion oil was used to surround the particles in a matching refractive index environment, and a cover glass with an anti-reflective coating for 1000–1300 nm wavelengths was placed on top to complete the sample. The anti-reflective coating was used to avoid Fabry–Pérot resonances from multiple reflections from different interfaces. For more information, see the Supporting Information.

**Figure 1:**
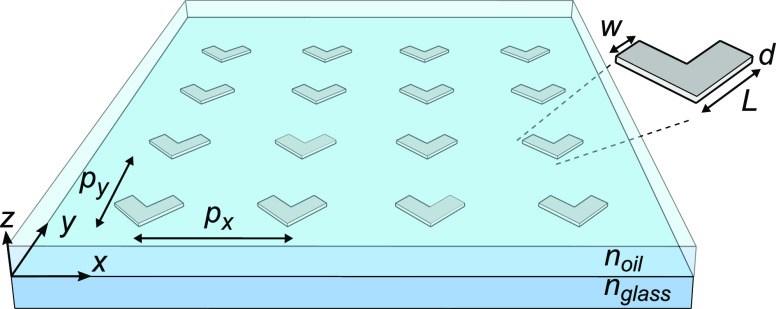
Investigated metasurface consisting of V-shaped aluminum NPs fabricated on a glass substrate (*n*_glass_ = 1.511) and covered by immersion oil. Here, *w* = 70 nm, *L* = 140 nm, and *d* = 30 nm are the arm widths, arm lengths, and the thicknesses of the NPs. The NPs were arranged in a rectangular lattice (*p*_*x*_ = 727 nm and *p*_*y*_ = 400 nm), giving rise to surface lattice resonances near 1100 nm for *y*-polarized light.

The above particle dimensions and the periodicity of 727 nm were chosen to place the SLR relatively far away from the LSPR. This approach allows one to considerably reduce the absorption and scattering losses associated with LSPRs and results in emergence of SLRs with relatively high *Q*-factors.^13^

The experimental setup is shown in Figure 2. A Fianium supercontinuum laser was used as a broadband light source with wavelength range of 300–2700 nm and maximum power < 50 W. The supercontinuum power was kept at a suitable level (<20 mW) using a neutral-density filter in order to keep the possible absorption-based heating of the sample negligible. Ambient *T* of the sample was controlled with a standard thermoelectric cooler, connected to an adjustable voltage source for fine-tuning of the temperature. The *T* of the sample was determined using a Flir E85 thermal imaging camera. A linear polarizer before the sample ensured the sample excitation using linearly polarized light. An iris after the sample was used to limit the collection of light to include only light passed through the desired metasurface array. Finally, the beam of light was guided to an optical spectrum analyzer through a single-mode optical fiber.

**Figure 2:**
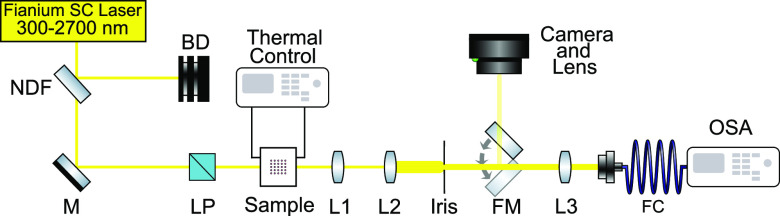
Schematic representation of the experimental setup used to measure the transmission spectra and to control the ambient temperature of the studied structures. SC, supercontinuum; NDF, neutral-density filter; BD, beam dump; M, mirror; LP, linear polarizer; L, lenses (L1, *f* = 19 mm; L2, *f* = 75 mm; L3, *f* = 4.6 mm); FM, flip mirror; FC, fiber coupler; OSA, optical spectrum analyzer; SM, spectrometer.

The measured transmission spectra with ambient temperature ranging from 11 to 39 °C are shown in Figure 3a. The results are in good agreement with those extracted from semianalytical LSA calculations (black lines). We note that some of the predicted transmission spectra show values exceeding unity, arising from the simplicity of the LSA approach. Here, unity-exceeding values correspond to a case where the scattered fields from nearby particles are strong enough to compensate the transmission losses associated with the effective polarizability of the NP. We also note that the LSA calculations are readily extendable to higher temperature ranges (see the Supporting Information for more details on LSA calculations).

**Figure 3:**
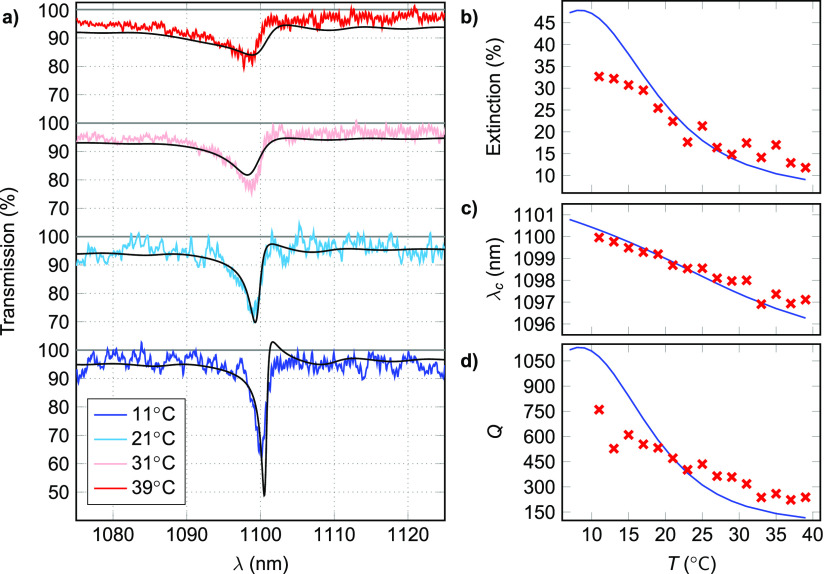
(a) Transmission spectra measured at different ambient temperatures, *T*. The black lines represent respective LSA calculations. The SLR peak at 39 °C with central wavelength of 1097 nm and a line width of 5 nm gets narrower and red-shifted with decreasing *T* down to a line width of 1 nm and central location of 1100 nm at 11 °C. (b) Peak extinction, (c) spectral location, λ_c_, and (d) *Q*-factor of the resonance as a function of *T*. The experimental data are marked with red crosses and the LSA calculations with solid blue lines.

The *Q*-factors, peak extinctions, and λ_c_ of the peak were obtained by fitting a Lorentzian line shape to the experimental data (see the Supporting Information for fitted models). The resonance showed a decreasing *Q*-factor and extinction, and a minor blue shift with increasing temperature. A linear regression for spectral location data showed an average decrease in λ_c_ of 0.11 nm/°C. This result can be attributed mainly to the decrease in the refractive index of the immersion oil with increasing *T*. Strikingly, the *Q*-factor and extinction both showed a 3.5-fold decrease in magnitude in the temperature range from 11 to 39 °C. The strong responsiveness to temperature is a result of symmetry breaking of the refractive index profile of the dielectric environment. The shift in peak location λ_c_ has also a minor effect on both the *Q*-factor and extinction as the energetic overlap between plasmonic and diffractive modes increases with rising temperature.

The behavior of the peak extinction, location, and *Q*-factor are shown in Figure 3b–d, with the experimental data marked with red. The extended temperature range of the LSA calculations in blue show that both the *Q*-factor and the extinction should reach their maximum values at around 8 °C. At this temperature, the NP surroundings become symmetric (*n*_oil_ = *n*_glass_). With increasing *T*, the refractive index of the index matching oil decreases faster (d*n*_oil_/d*T* = −3.3 × 10^–4^ 1/°C) than that of the glass substrate (d*n*_glass_/d*T* ≈ – 6 × 10^–6^ 1/°C), resulting in symmetry breaking of the environment. We expect the difference in the refractive indices (*n*_glass_ – *n*_oil_) to range from 0 at around 8 °C to around 1 × 10^–2^ at 40 °C in an almost linear manner. The effects of symmetry breaking in the *Q*-factor and extinction are therefore confirmed by the LSA calculations (see Figure 3b,d and the Supporting Information).

While the *Q*-factor and extinction of the SLR are expected to rise even more with lower temperatures down until 8 °C, the humidity of our laboratory limited our measurements to 11 °C. At colder temperatures water condensation to the sample surfaces made further experiments unfeasible. Interestingly, prior theoretical work^29^ seems to suggest that symmetry breaking affects the SLR strength only when the substrate is optically denser than the superstrate. This would imply that the extinction would not diminish at temperatures below 8 °C in our experiments. We, however, expect the *Q*-factor to decrease in temperatures lower than 8 °C due to symmetry breaking. In the future, it would therefore be interesting to study the behavior experimentally. Nevertheless, our results still show dramatic changes in the SLR properties already with quite small changes in the ambient temperature. Furthermore, we operate the devices close to room temperatures, which demonstrates that the approach could be an easily and broadly applicable tuning method.

Our results confirm the significance of a symmetric environment in high-*Q* SLR metasurfaces. We estimate that already a modest change of ∼0.003 units in the refractive indices of the super- and substrate (from temperatures 21 to 11 °C) raised the *Q*-factor of the resonance from 400 to 750. Furthermore, the results show that the spectral properties of SLRs can be efficiently controlled via symmetry breaking in the sample, which is particularly interesting when noting that alternative means of changing the refractive index of the superstrate could be used in a manner similar to the temperature control. An ultrafast alternative could be to utilize Kerr-active materials, making it possible to control the properties of the SLRs by using an external voltage source or by using a control beam of light. For example, a superstrate made of Kerr-active As_2_S_3_ chalcogenide glass (*n*_2_ = 2 × 10^–13^ cm^2^/W) could provide a change of refractive index similar to what was demonstrated here (∼0.003) using a control beam with a moderate 15 GW/cm^2^ peak intensity. We note that even smaller changes in refractive index profiles would be needed, if one would utilize this/similar approach to modulate narrowband (<1 nm) light sources or could utilize SLRs associated with even higher *Q*-factors than what the investigated structures exhibited.

To conclude, we have investigated how spectral properties of plasmonic surface lattice resonances can be modified by controlling the ambient temperature of the studied metasurface. Our metasurface consisted of aluminum nanoparticles arranged in a rectangular array on a glass substrate covered by immersion oil. At room temperature, the metasurface exhibited a high quality factor (*Q* ≈ 400) SLR near 1100 nm. By decreasing the ambient temperature by 10 °C, the SLR peak was slightly red-shifted and the *Q*-factor was increased to 750. The increased *Q*-factor is explained by the improved symmetry of the nanoparticle surroundings, resulting from the temperature-dependent refractive index of the overlying immersion oil. Our results show that even slight changes in the refractive indices of the surrounding materials can result in dramatic changes in the SLR properties, simultaneously demonstrating their accurate and reversible tunability.

## Supporting Information Available

Details regarding sample fabrication; descriptions of model used for localized surface plasmon resonances and of lattice-sum approach calculations; temperature effects on bulk metal permittivity; extraction of peak parameters from experimental and simulated extinction data (PDF)
