# Supplementary material for: Thermal Control of Plasmonic Surface Lattice Resonances
Source: Nano Lett. 2022 May 4;22(10):3879–83. doi: 10.1021/acs.nanolett.1c04898 (PMC9136927; doi:10.1021/acs.nanolett.1c04898)
Supplement: Supplementary file 1 — nl1c04898_si_001.pdf [file nl1c04898_si_001.pdf]

Supporting Information for Thermal Control of Plasmonic Surface Lattice Resonances

Jussi Kelavuori,[†] Viatcheslav Vanyukov,[‡] Timo Stolt,[†] Petri Karvinen,[‡] Heikki Rekola,[‡] Tommi K. Hakala,[‡] and Mikko J. Huttunen[*,†]

†*Photonics Laboratory, Physics Unit, Tampere University, FI-33014 Tampere, Finland*

‡*Faculty of Science and Forestry, Department of Physics and Mathematics, University of Eastern Finland, FI-80101 Joensuu, Finland*

E-mail: mikko.huttunen@tuni.fi

Sample Fabrication

For this work, we fabricated V-shaped aluminum (Al) nanoparticle arrays with a total area of 300×300 µm^2. The structures were fabricated on a pre-cleaned microscope slide (Schott Nexterion, D263T glass). A 200 nm layer of PMMA-resist (MicroChem, 950k) was spin-coated on top and baked on a hot plate at 180°C for 180 s. A 10 nm layer of Al was evaporated on the resist to act as a conductive layer for electron beam lithography.

The patterning was done using a Raith EBPG 5000+ 100 kV electron beam lithography system. After patterning the Al layer was removed using a 1% sodium hydroxide solution. The resist was then developed using a 1:3 mixture of methyl isobutyl ketone and isopropanol (IPA) for 15 s, followed by a 30 s immersion in IPA. The sample was dried with nitrogen and placed in an electron beam evaporator for depositing 30 nm of Al. Finally, a liftoff process was performed by soaking the sample in acetone overnight and gently washing the surface with more acetone using a syringe. This removes the resist and excess metal on top of it,

leaving only the nanoparticles on the glass substrate. The sample was then rinsed with IPA and dried with nitrogen.

Before the measurements, we covered the metasurface with index-matching oil and an anti-reflection (AR) coated coverslip with the AR wavelength band at 1000–1300 nm. This way, the nanoparticles were assured to have a homogeneous surrounding, and we avoided any Fabry–Pérot resonances resulting from multiple reflections from different interfaces present in the fabricated devices.

Localized Surface Plasmon Resonance

Polarizability for NPs of arbitraty shape is given by[1]

$$\alpha_i = V \frac{\varepsilon_m - \varepsilon_s}{\varepsilon_s + L_i(\varepsilon_m - \varepsilon_s)}, \tag{1}$$

where V is the volume of the nanoparticle (NP), ε_m and ε_s are the permittivities of the metal and surrounding media respectively, L_i is a factor depending on the geometry of the particle, and index i marks the relevant dimension. While L_i has been analytically solved for shapes such as slabs and ellipsoids,[1] a general analytical solution for complicated shapes, such as the V-shaped particles used in this work, does not exist. Consequently, we used a value of $L_y = 0.26$, that was found by trial and error to match well with the experiments.

Equation (1) assumes the electric field to be static at a given time, which produces accurate results while the dimensions of the NPs are smaller than 1% of the incident wavelength.[2] With our NPs, however, a modified long-wavelength approximation (MLWA) using dynamic perturbations must be used for accurate modelling of the localized surface plasmon resonances (LSPRs). After applying MLWA, polarizability can be written as[3]

$$\alpha_{i,\mathrm{MLWA}} = \frac{\alpha_i}{1 - \mathrm{i}\frac{k^3}{6\pi}\alpha_i - \frac{k^2}{4\pi a_i}\alpha_i}, \tag{2}$$

where α_i is the static polarizability, and k is the wavenumber of the incident field.

Figure 1 shows experimental data next to analytical model using equations (1) and (2). The experimental data is measured from the same metasurface as the SLRs in this work. It should be noted that the LSPR is not pure due to the presence of the second order diffraction-mode near 550 nm. In the equation (1), experimental data for aluminum permittivity from Rakić[4] was used for ε_m. The surroundings were modelled using the permittivity of the SCHOTT - multiple purpose D 263® T eco Thin glass. In the Equation (2), a NP dimension of $a_y = 75$ nm was used, corresponding approximately to half of the length of the NP in y-direction. In Equation (1) a geometrical factor a of $L_y = 0.26$ was used.

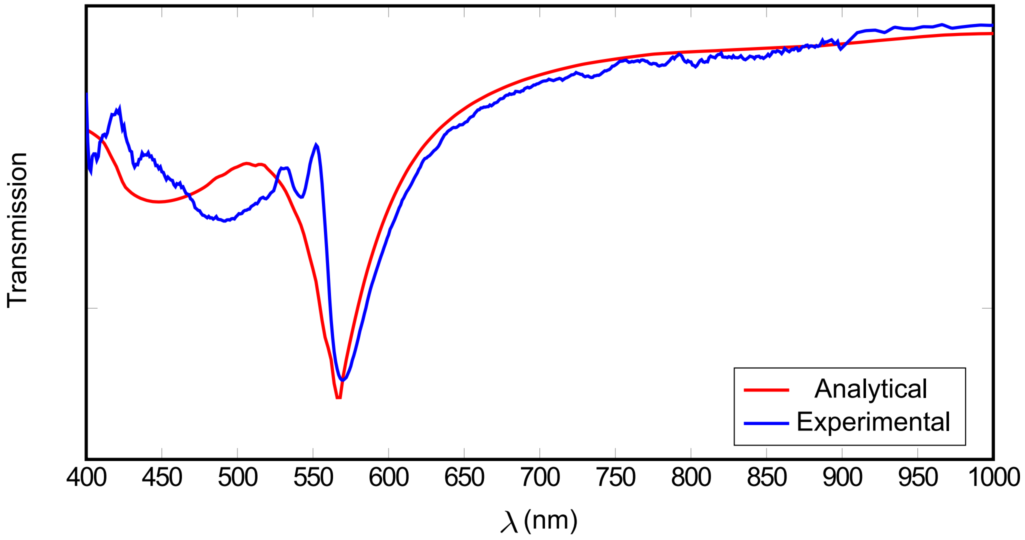

Figure 1: Measured LSPR of the metasurface used in the work (blue). The LSPR given by the LSA calculations used in this work (red).

Lattice Sum Approach

Lattice sum approach (LSA) method was used to support the experimental results with semianalytical calculations. LSA is a simplified version of the discrete-dipole approximation (DDA) method that allows to calculate the response of optically coupled nanoparticles. An interested reader is referred to Ref. [5] for further details regarding the similarities and

differences between the LSA and the DDA approaches. Shortly put, in LSA the effects of coupling between nanoparticles are reduced to a single constant for all nanoparticles. The reduction is done with the loss of generality in non-infinite lattices, with LSA being unable to model dipole moments of the nanoparticles near the edges of the lattice. The LSA is based on several assumptions. Most importantly, all the nanoparticles are treated as point-like objects which scatter the incoming light as electric dipoles. In LSA specifically, it is assumed that all the nanoparticles are identical, and experience identical environments with respect to each other.

In LSA, a concept of effective polarizability α^* is introduced, which associates the effectively induced dipole moments $\mathbf{p}$ to the incident electric field $\mathbf{E}$ via $\mathbf{p} = \varepsilon_0 \varepsilon_s \alpha^* \mathbf{E}$, where ε_0 and ε_s are the vacuum permittivity and relative permittivity of the surroundings, respectively. The effective polarizability is given by

$$\alpha_i^* = \frac{1}{1/\alpha_i - S_i}, \tag{3}$$

where i denotes a component of the quantity in Cartesian direction, α_i is the single particle polarizability, and S_i is the lattice sum.

The lattice sum in equation (3) can be expressed in a homogeneous environment as[5]

$$S_i(k) = \sum_{j=1}^{N} \frac{\mathrm{e}^{\mathrm{i}kr_j}}{4\pi r_j} \left[k^2 \sin^2 \varphi_{i,j} + \frac{(1 - \mathrm{i}kr_j)\,(3\cos^2 \varphi_{i,j} - 1)}{r_j^2} \right], \tag{4}$$

where N is the number of particles taken into account, r_j is the distance between j^{th} particle and the center particle, and $\varphi_{i,j}$ is the angle between the ith dipole moment component and the vector from the center particle to j^{th} particle. The lattice sum S can be derived from DDA by assuming identical dipole moments for all NPs.[5]

In this work, the studied NP arrays lied on an interface between Olympos IMMOIL-F30CC -immersion oil and SCHOTT - multiple purpose D 263® T eco Thin Glass. This caused slight heterogeneity in the surroundings of the SLR at room temperature. To model

the heterogeneity, the coupling of NPs was thought to develop independently in the different materials. We assumed the heterogeneous lattice sum to be a weighted mean of two different homogeneous lattice sums arising in the different surrounding materials. Changing the weights make the peak location to shift continuously between the two homogeneous peak locations. Therefore, the weights could be determined by comparing the shift in the peak location in the LSA calculations to the ones extracted from experimental results. The analysis arrived at even weights for both materials. Therefore the heterogeneous lattice sum is calculated in our case as follows

$$S_{i,\mathrm{het}}(k_{\mathrm{oil}}, k_{\mathrm{glass}}) = \frac{S_i(k_{\mathrm{oil}}) + S_i(k_{\mathrm{glass}})}{2}, \tag{5}$$

where k_{oil} and k_{glass} are the wavenumbers in the (Olympos IMMOIL-F30CC -immersion) oil and (SCHOTT - multiple purpose D 263® T eco Thin) Glass respectively, given by

$$k_{\mathrm{oil}} = \frac{2\pi n_{\mathrm{oil}}(\lambda, T)}{\lambda}, \qquad k_{\mathrm{glass}} = \frac{2\pi n_{\mathrm{glass}}(\lambda, T)}{\lambda} \tag{6}$$

where λ is the wavelength, and $n_{\mathrm{oil}}(\lambda, T)$ and $n_{\mathrm{glass}}(\lambda, T)$ are the refractive indices of the oil and glass respectively. Dispersion data for $n_{\mathrm{glass}}(\lambda, T)$ was taken from.[6] With the lack of full description of temperature dependence in SCHOTT - multiple purpose D 263® T eco Thin Glass, a temperature dependence of a glass with similar properties (SCHOTT, N-KF9) was used.[7] $n_{\mathrm{oil}}(\lambda, T)$ was calculated from the constants $\mathrm{d}n/\mathrm{d}T = -3.3 \times 10^{-4}$ and Abbe number $V_e = 41$ given by the manufacturer.[8] With the immersion oil not designed to operate at infrared wavelengths, we added a free parameter to match the calculations better with experiments in longer wavelengths. Namely a constant of $n_0 = 0.035$ was added to the refractive index as follows: $n_{\mathrm{oil,used}}(\lambda, T) = n_{\mathrm{oil}}(\lambda, T) + n_0$.

It should be noted that while in both surrounding materials the refractive index decreases with increasing temperature, the effect is roughly one magnitude stronger in the oil superstrate compared to the glass substrate. This discrepancy causes the heterogeneity in the

environment of the NPs to rapidly rise with increasing temperature. In the calculations this shows up first as widened peaks in both the real and imaginary parts of the lattice sum $S_{i,\text{het}}$ near the diffraction orders associated with the lattice. At high enough temperatures, two separate peaks will form, proving the method demonstrated by the equation (5) insufficient for modelling high heterogeneity in the surroundings of the NPs. However, good correlation between the performed LSA calculations and the experimental data was observed in the narrow temperature range studied in our work.

While the heterogeneity of the surroundings is not the only factor affecting the SLR, it is the most important one regarding Q-factor and extinction. Other factors affecting the SLR-properties include the energetic overlap between the plasmonic and diffractive modes, with higher Q-factors effectively achieved with smaller values of polarizability α at the diffraction mode wavelength. Ways to modify the polarizability α include control over the size, shape and material of the NP, and control over the refractive index of the surrounding medium (see Equation (1)). Post-fabrication control over the single NP properties therefore usually depend on changing the refractive index of the surrounding medium (such as in biosensing with plasmonic NPs[9]). Additionally, temperature has an effect on the permittivity of the metal, and therefore on the single NP properties. These effects have been investigated in the following section.

Effects of temperature on the permittivity of the metal

It is difficult to model the temperature dependence of the aluminum permittivity using the Drude-model near visible and infra-red frequencies due to interband excitations near 800 nm. However, since the experiments were carried out near the wavelength of 1100 nm, the interband excitations taking place near 800 nm can be expected to play a negligible role. Nevertheless, we decided to estimate the temperature dependence of the permittivity of metallic NPs, and subsequent changes in the SLRs of periodic nanoparticle arrays, for gold

NPs. This approach was chosen because the permittivity of aluminum can be expected to behave very similarly to that of gold near the wavelength of 1100 nm. The permittivity of gold was modelled using the Drude model:[10]

$$\varepsilon_m(T) = \varepsilon_\infty - \frac{\omega_p^2(T)}{\omega(\omega + i\gamma(T))}, \tag{7}$$

where ε_m is the permittivity of gold, ε_∞ is the high-frequency permittivity, ω_p is the plasma frequency of the metal, ω is the angular frequency of the incident light, and γ is the relaxation constant for the free electrons. Plasma frequency is given by[10]

$$\omega_p(T) = \sqrt{\frac{n_o e^2}{\varepsilon_0 m(1 + 3\beta\Delta T)}}, \tag{8}$$

where n_0 is the free electron number density in the metal, e is the elementary charge, ε_0 is the vacuum permittivity, m is the mass of electron, β is the linear thermal expansion coefficient and $\Delta T = T - T_0$ is the difference to the temperature of comparison T_0. The term $1 + 3\beta\Delta T$ arises from the thermal expansion of the material, which is the temperature dependent effect affecting the plasma frequency.

The thermal relaxation constant γ is comprised of electron–electron contribution $\gamma_{\mathrm{e-e}}(T)$ and electron–phonon contribution $\gamma_{\mathrm{e-ph}}(T)$, which are given by[10]

$$\begin{aligned} \gamma_{e-e}(T) &= A\left[(k_B T)^2 + (\hbar\omega)^2\right], \\ \gamma_{e-ph}(T) &= \gamma_0 \left[\frac{2}{5} + \frac{4T^5}{\theta_D^5} \int_0^{\theta_D/T} \frac{x^4}{e^x - 1}\, \mathrm{d}x\right], \end{aligned} \tag{9}$$

where the factor A is dependent on the properties of the conduction band of the metal,[10] k_B is the Boltzmann constant, $\hbar$ is the reduced Planck's constant, γ_0 is the temperature-independent electron–phonon relaxation constant and Θ_D is the Debye-temperature. $\varepsilon_\infty = 11.5$ and $\hbar\gamma_0 = 0.07\,\mathrm{eV}$ were determined by fitting gold bulk permittivity (from Johnson & Christy[11]) to the model. Other constants for gold are $n_0 = 5.9 \times 10^{28}\,\mathrm{m}^{-3}$, $\beta =$

$14.2 \times 10^{-6}\,\mathrm{K}^{-1}$,,[12] $\hbar A = 0.0317\,\mathrm{eV}^{-1}$,[10] and $\Theta_D = 170\,\mathrm{K}$.[13]

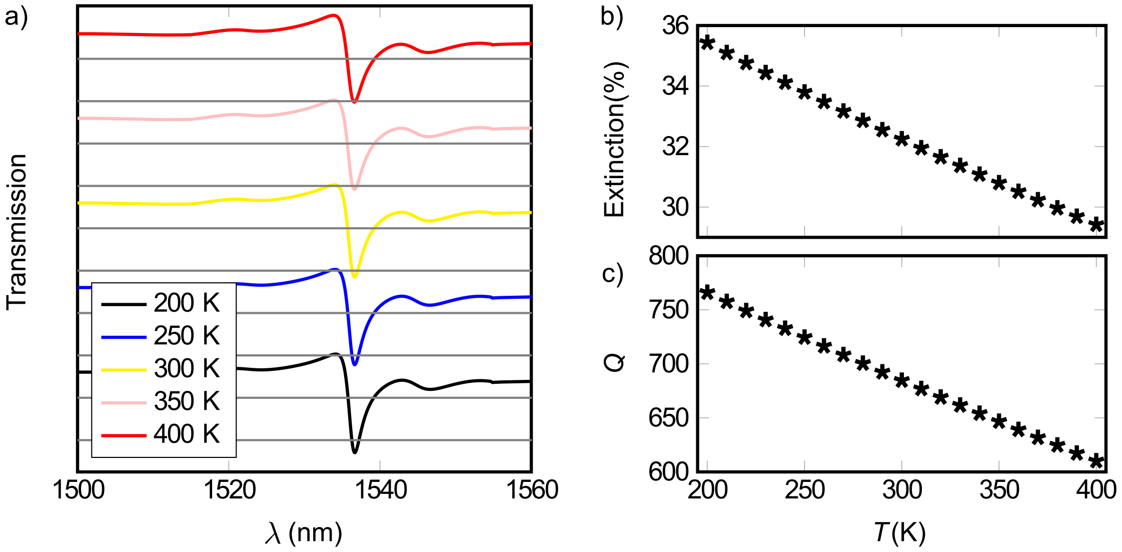

Figure 2: a) The transmission spectra calculated using LSA for homogeneous, temperature independent surroundings for temperature dependent gold NPs. A horizontal line represents a drop of 20 % in transmittance. The extinction b) and Q-factor c) of the SLR in different temperatures. The spectral location of the peak hardly showed any change with temperature.

The resulting temperature-dependent permittivity was used in the Equation (1) for polarizability α, which in turn was used in the LSA calculations to estimate how the temperature dependence of the permittivity affects the formation of the SLR. The results are shown in figure 2. Even with a broad temperature range used in the calculations ($\Delta T = 200$ K), the line shapes of the SLRs were almost unnoticeably modified.

It is evident that the slight temperature dependence of the permittivity of the metal has little effect on the SLR in the small temperature range ($\Delta T = 30$ K) we used in our work. Attempts to model the effects of the temperature dependence of aluminum were therefore omitted. However, we note that in the case of perfectly homogeneous environment, the intrinsic temperature dependence of the metal could significant change the formation of the SLR, when broad enough temperature ranges are used. In particular, looking at our results, it is evident that the Q-factors of the SLRs could be increased when samples would be cooled to cryogenic temperatures. Other effect affecting SLRs in homogeneous environments is the shifting of the spectral location of the SLR with temperature dependent refractive index of

the homogeneous substrate.

Extraction of peak parameters from experimental and LSA data

All Q-factors, peak locations λ_c and extinctions were determined in this work by fitting a Lorentzian line shape to the extinction spectra. Lorentzian is a line shape given by equation

$$f(\lambda; \lambda_c, \gamma_0, I) = I \left[\frac{\gamma^2}{(\lambda - \lambda_c)^2 + \gamma^2} \right], \tag{10}$$

where λ is the wavelength, I is the peak extinction of the resonance peak, and γ_0 is the half width at half maximum. The Q-factor is therefore given by

$$Q = \frac{\lambda_c^{-1}}{(\lambda_c + \gamma_0)^{-1} - (\lambda_c - \gamma_0)^{-1}}. \tag{11}$$

The fitted models are shown along with the experimental data on Figure 3.

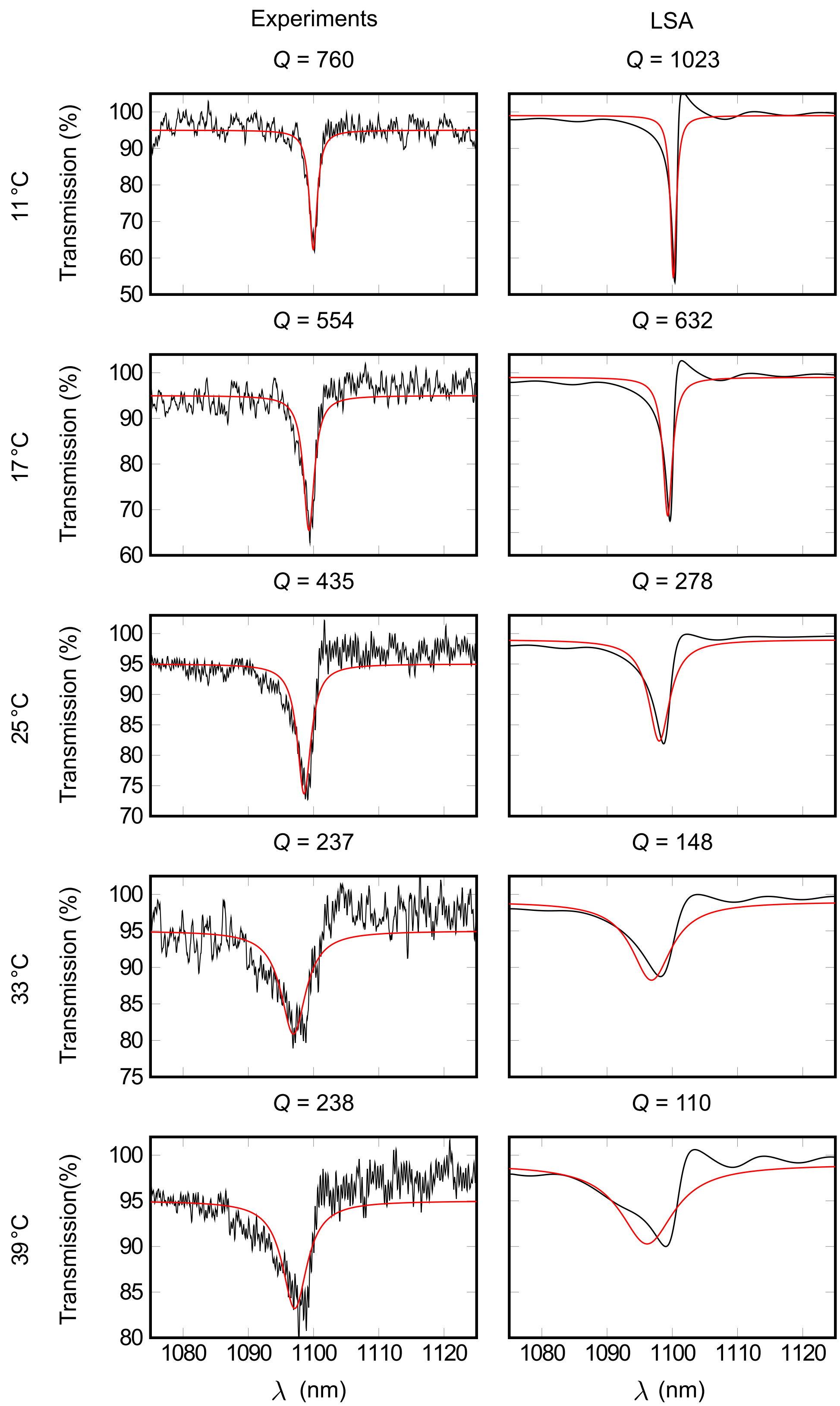

Figure 3: Fitted Lorentzian lineshapes (red) on top of experimental data (black). Take note of the different y-axes for different temperatures.

References

(1) Barnes, W. L. Particle plasmons: Why shape matters. **2016**,

(2) Jensen, T.; Kelly, L.; Lazarides, A.; Schatz, G. C. Electrodynamics of noble metal nanoparticles and nanoparticle clusters. *Journal of Cluster Science* **1999**, *10*, 295–317.

(3) Zorić, I.; Zäch, M.; Kasemo, B.; Langhammer, C. Gold, platinum, and aluminum nanodisk plasmons: Material independence, subradiance, and damping mechanisms. *ACS Nano* **2011**, *5*, 2535–2546.

(4) Rakić, A. D. Algorithm for the determination of intrinsic optical constants of metal films: application to aluminum. *Appl. Opt.* **1995**, *34*, 4755–4767.

(5) Huttunen, M. J.; Dolgaleva, K.; Törmä, P.; Boyd, R. W. Ultra-strong polarization dependence of surface lattice resonances with out-of-plane plasmon oscillations. *Optics Express* **2016**, *24*, 28279–28289.

(6) SCHOTT Zemax catalog 2017-01-20b. 2017; `https://refractiveindex.info/download/data/2017/schott_2017-01-20b.agf`.

(7) Optical Glass, Data Sheets. 2017; `http://refractiveindex.info/download/data/2017/schott_2017-01-20.pdf`.

(8) Abramowitz, M.; Davidson, M. W. Microscope Optical Components Immersion Media. `https://www.olympus-lifescience.com/en/microscope-resource/primer/anatomy/immersion/`, Read: 13.7.2021.

(9) Unser, S.; Bruzas, I.; He, J.; Sagle, L. Localized surface plasmon resonance biosensing: Current challenges and approaches. *Sensors (Switzerland)* **2015**, *15*, 15684–15716.

(10) Bouillard, J. S. G.; Dickson, W.; O'Connor, D. P.; Wurtz, G. A.; Zayats, A. V. Low-temperature plasmonics of metallic nanostructures. *Nano Letters* **2012**, *12*, 1561–1565.

(11) P. B. Johnson and R. W. Christy,; Johnson, P. B.; Christy, R. W. Optical constants of the noble metals. *Physical Review B* **1972**, *6*, 4370–4379.

(12) CRC Handbook, *CRC Handbook of Chemistry and Physics, 88th Edition*, 88th ed.; CRC Press, 2007.

(13) Kittel, C. *Introduction to Solid State Physics*; Wiley, 2004.
